# Extracellular matrix: the critical contributor to skeletal muscle regeneration—a comprehensive review

**DOI:** 10.1186/s41232-023-00308-z

**Published:** 2023-11-27

**Authors:** Khurshid Ahmad, Sibhghatulla Shaikh, Hee Jin Chun, Shahid Ali, Jeong Ho Lim, Syed Sayeed Ahmad, Eun Ju Lee, Inho Choi

**Affiliations:** 1 Department of Medical Biotechnology, Yeungnam University, Gyeongsan, 38541 South Korea; 2 Research Institute of Cell Culture, Yeungnam University, Gyeongsan, 38541 South Korea

**Keywords:** Extracellular matrix, Skeletal muscle, Regeneration, Muscle loss, Biomaterials, ECM scaffold

## Abstract

The regenerative ability of skeletal muscle (SM) in response to damage, injury, or disease is a highly intricate process that involves the coordinated activities of multiple cell types and biomolecular factors. Of these, extracellular matrix (ECM) is considered a fundamental component of SM regenerative ability. This review briefly discusses SM myogenesis and regeneration, the roles played by muscle satellite cells (MSCs), other cells, and ECM components, and the effects of their dysregulations on these processes. In addition, we review the various types of ECM scaffolds and biomaterials used for SM regeneration, their applications, recent advances in ECM scaffold research, and their impacts on tissue engineering and SM regeneration, especially in the context of severe muscle injury, which frequently results in substantial muscle loss and impaired regenerative capacity. This review was undertaken to provide a comprehensive overview of SM myogenesis and regeneration, the stem cells used for muscle regeneration, the significance of ECM in SM regeneration, and to enhance understanding of the essential role of the ECM scaffold during SM regeneration.

## Introduction

Skeletal muscle (SM) is a highly coordinated tissue composed of myofibers formed by myogenic progenitor cell fusion [1] and is primarily responsible for skeletal support, body movement, and temperature regulation. Furthermore, SM has an impressive ability to regenerate after injury, and this ability depends on resident muscle satellite (stem) cells (MSCs) located in unique anatomical sites along the margins of myofibers [2]. MSCs dynamically regulate the development and progression of myofibers using several transcription factors that serve as key regulators of the quiescent state and activators of progression to the myogenic lineage [3].

Regeneration is an essential process in living organisms because tissues and organs are all susceptible to damage, and thus, mechanisms responsible for their repair must be in good working order to conserve physiology and function [4]. Multiple cell types, especially MSCs, are involved in the SM regeneration triggered in response to damage, injury, or disease [5, 6]. When a muscle is injured, MSCs are activated, transform into myoblasts, and fuse to produce myotubes, which eventually develop into new muscle fibers [7]. The three phases of SM regeneration are the inflammatory/destructive, healing, and remodeling phases [8].

The ECM is a complex assembly that supports cells mechanically, sustains biochemical signaling, and acts as a key player in SM regeneration [9]. ECM components interact with respective cell receptors and regulate proliferation, migration, and differentiation processes [10]. ECM molecules and transmembrane receptors aid muscle contraction, growth, regeneration, and development [11]. Acellular ECM and biological scaffolds of ECM proteins have been used to regenerate tissues like cardiac muscles, SM, and abdominal skin [12]. Furthermore, extensive research has been conducted on the use of acellular ECM scaffolds, especially in combination with progenitor cells, for treating severe muscle mass damage (known as volumetric muscle loss; VML) [13]. The destruction/removal of the basal lamina and the loss of other structural muscle components, such as MSCs, are the greatest constraints to VML regeneration [14]. Repairing SM damage caused by VML is a complex and challenging process that involves multiple stages. ECM scaffolds offer a potential clinically applicable regenerative biomaterial that can aid this process as they are easily obtained, inexpensive, and have the potential to improve muscle regeneration, all of which make them excellent materials for VML repair [15].

Several ECM components, including fibromodulin (FMOD) [16–18], matrix gla protein (MGP) [19], and dermatopontin (DPT) [20], have been linked to the regulation of myogenesis and the promotion of SM regeneration. In a recent study on the role of the IgLON family in myogenesis and SM regeneration, we found IgLON4 promotes myogenesis and regeneration by enhancing cell adhesion and maintaining myotube orientation [21] and that IgLON5, which helps in myoblast adhesion and differentiation, is essential for myogenesis and regeneration [22]. For the present review, we accessed over 500 articles, including original research and review papers related to SM regeneration published in peer-reviewed journals from 2012 to Feb 2023, using the search terms “skeletal muscle regeneration”, “skeletal muscle extracellular matrix”, “ECM scaffold”, and “volumetric muscle loss”. We aimed to provide an in-depth understanding of the complex mechanisms involved in the formation and repair of SM with a focus on the types of stem cells involved in the process. In addition, we reviewed the essential roles of ECM in normal muscle and severely damaged muscle under VML conditions and in SM regeneration and recent developments. This review was undertaken to advance our understanding of the role of ECM in muscle regeneration and provide insights into the most recent research conducted on the topic.

### Skeletal muscle formation and regeneration

**Fig. 1:**
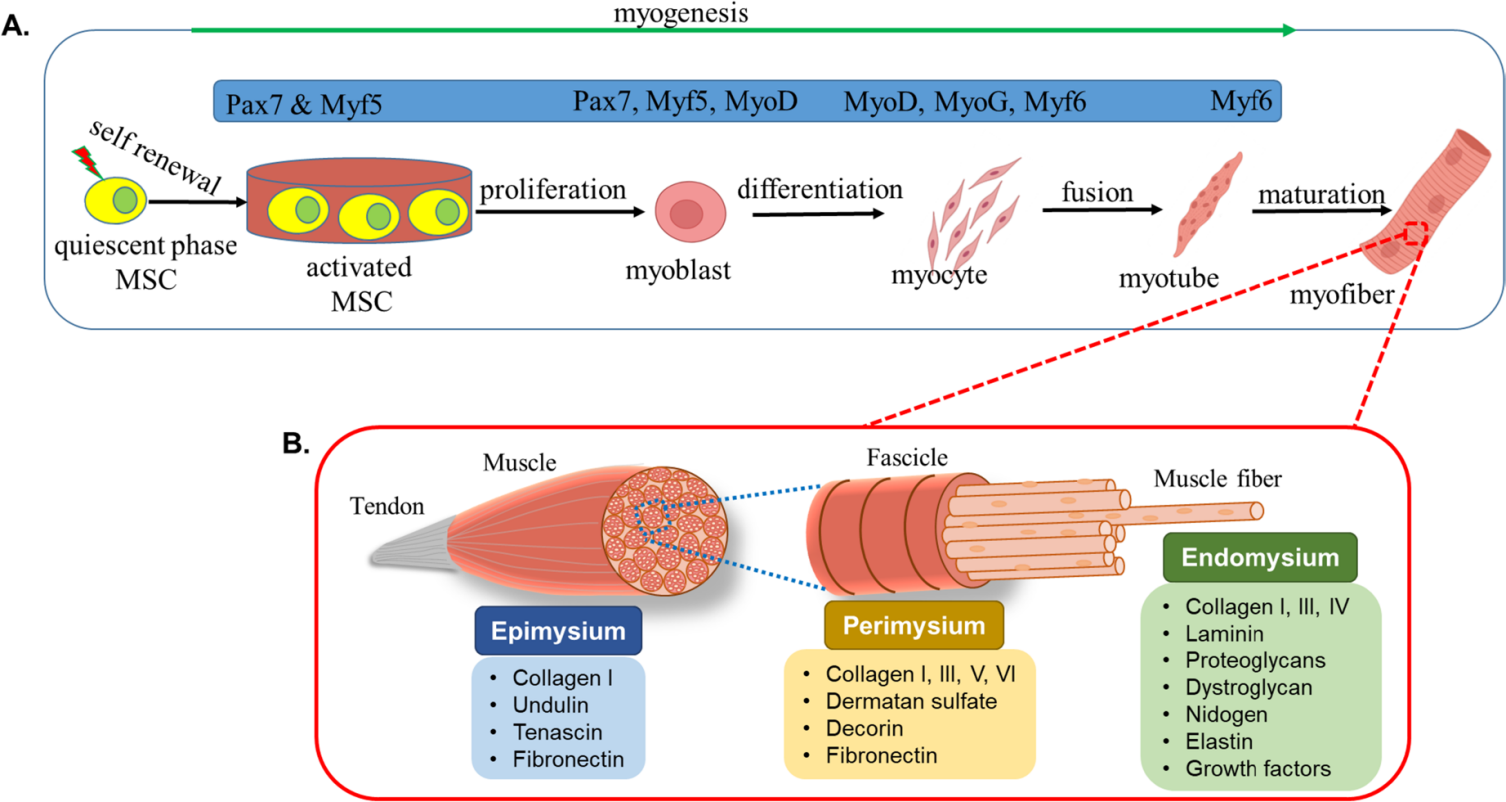
SM regeneration: **A** muscle regulatory factors at the different stages of myogenesis, **B** three distinct layers of the SM (epimysium, perimysium, and endomysium) and associated ECM components

SM is essential for movement, postural support, and stability [23]. Typically, muscle mass maintenance depends upon the balance between the rates of protein synthesis and breakdown, and this balance is influenced by several factors, including dietary status, hormonal balance, physical activity/exercise, injury, and disease [1]. Myogenesis is the process of creating new muscle cells and fibers, and this process occurs in three stages, viz. embryonic myogenesis, secondary myogenesis, and postnatal myogenesis, that sometimes overlap [24]. During embryonic myogenesis, primary myotubes are formed and the fundamental muscle architecture is established, which are both crucial for the development and organization of muscle tissue [25]. Later during embryonic development, secondary myogenesis results in the formation of secondary myofibers, which constitute most muscle mass present at birth. The postnatal phase is mediated by MSCs and is responsible for postnatal growth and muscle regeneration [26–28]. These phases are controlled primarily by the myogenic regulatory factors (MRFs), Myf5, MyoD, myogenin, and MRF4/Myf6, which are commonly referred to as the “master” transcription factors that govern the regulation of SM development and differentiation [29, 30] (Fig. 1).

In mice, embryonic SM development begins around embryonic day 10.5 (E10.5) and depends on PAX3^+^ muscle progenitors. The initial development of muscle fibers occurs when PAX3 + /PAX7 + progenitor cells within the myotome undergo differentiation, which involves the activations of MRFs and leads to the differentiation and fusion of these progenitor cells. Fetal myogenesis begins later at about E14.5 in the mouse; muscle progenitors that express PAX7 differentiate into myoblasts that merge with pre-existing embryonic fibers to enable muscle development [31]. Several signaling pathways are involved in the transition of SM progenitor cells from proliferation to differentiation. The NOTCH signaling system is a significant regulator of the muscle progenitor pool and reportedly suppresses myoblast differentiation in various animal models [32–34]. The proliferation state of muscle progenitors is also maintained by BMP signaling [35]. The involvement of WNT signaling during myogenesis differs across embryonic and fetal stages. WNT is not required for muscle progenitor or fiber development in the developing limbs of mice, but fetal progenitor expression of a constitutively active form of ß-catenin, an effector of the WNT pathway, enhances PAX7^+^ cell numbers [36].

**Fig. 2:**
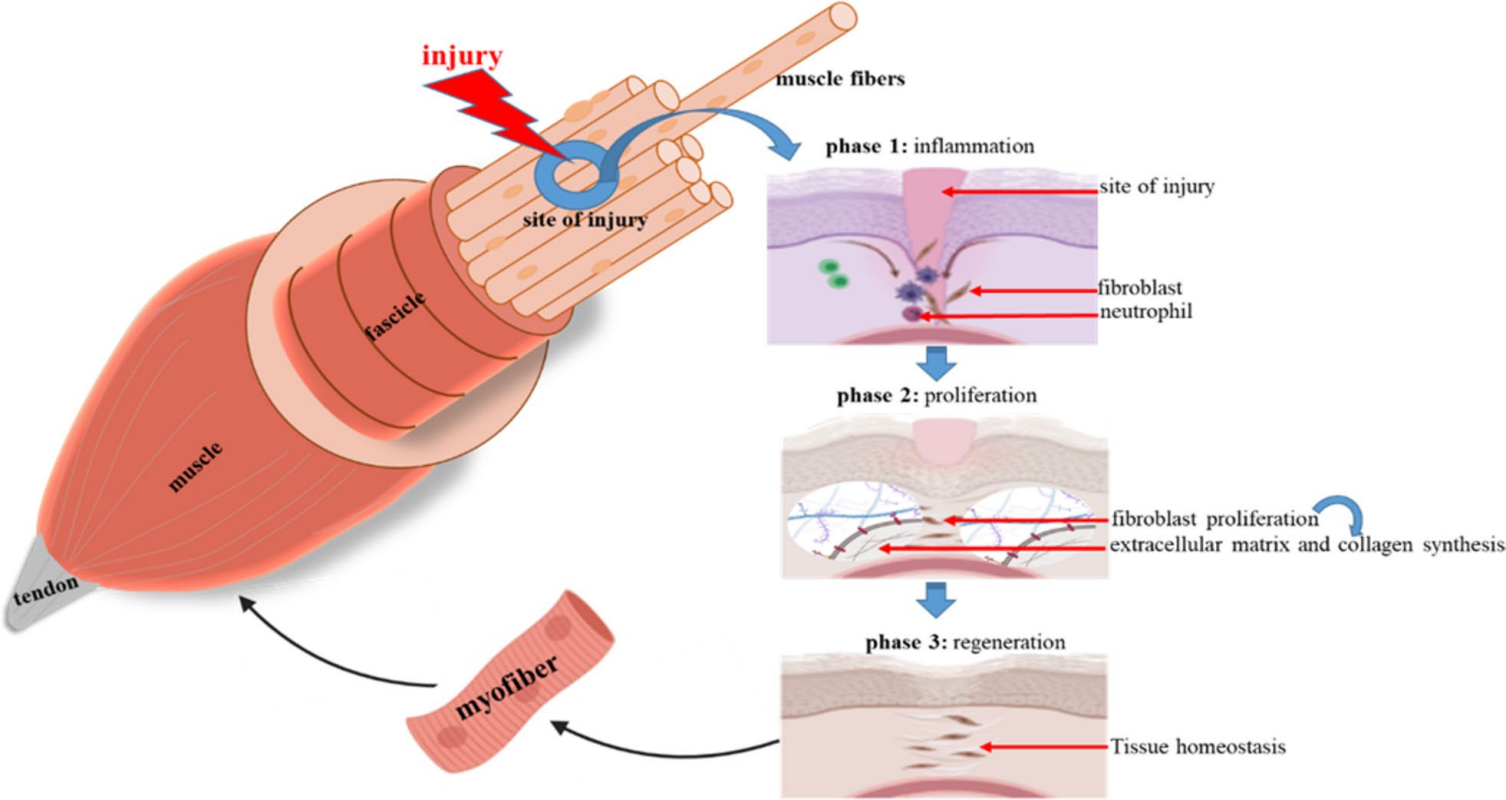
Illustration depicting the three distinct stages of SM regeneration following injury, including inflammation at the site of injury, fibroblast proliferation, formation and maturation of new fiber, and regeneration

SM possesses a notable ability to regenerate after injuries that substantially alter ECM and muscle cells [37]. Muscle regeneration after injury appears to be similar to muscle formation during embryogenesis and is regulated by various mechanisms, which involve cell–cell and cell–matrix interactions and the extracellular secretions of various factors [37, 38]. SM regeneration involves three phases: inflammation-induced destruction during the early phase, regeneration with myogenic cell activation to proliferate and differentiate, resulting in the formation of new myofibers, and finally, the reconstitution of a functional contractile apparatus [39] (Fig. 2). Muscle regeneration typically begins within the first week of injury, peaks at 2 weeks, and then gradually subsides [40]. Several types of cells have been associated with SM myogenesis and regeneration [41, 42], but MSCs are the primary repository of adult muscle regeneration. Some important cell types are described below and summarized in Table 1.

**Table 1:** Role of MSCs in muscle regeneration across various conditions and the current state of techniques/therapies

System/application	Role of MSCs in regeneration	Techniques/therapies	Reference
SM injury	MSCs proliferate and differentiate into new myofibers, promoting muscle tissue repair	Stem cell therapy, gene therapy, tissue engineering	[43]
Exercise-induced muscle damage	MSCs aid in repairing exercise-induced muscle damage, leading to muscle growth and adaptation	Activation of endogenous MSCs or transplantation of exogenous MSCs	[44, 45]
Aging	The reduction in MSCs number and regenerative ability partly contributes to age-related decline in muscle function	Exercise intervention, nutritional intervention, stem cell therapy	[46, 47]
Tissue engineering	MSCs differentiate into myoblasts and fuse to form new muscle fibers, enabling the repair of damaged or lost muscle tissue	Bioprinting, scaffold-based engineering, microfluidic devices	[48, 49]
Neuromuscular diseases	MSCs aid in regenerating muscle fibers in different neuromuscular conditions, including ALS and spinal muscular atrophy	Transplantation of MSCs directly into affected muscles or systemic delivery of MSCs	[50, 51]
Cancer cachexia	MSCs experience reduced regenerative capacity and contribute to muscle wasting	Nutritional intervention, exercise intervention, stem cell therapy, co-culture of MSCs with supportive cells	[52]
Diabetic myopathy	MSCs may play a role in the development of diabetic myopathy and are potential therapeutic targets for muscle regeneration in diabetes	Exercise intervention, nutritional intervention, stem cell therapy, co-culture of MSCs with supportive cells	[53, 54]
Congenital myopathies	MSCs contribute to muscle regeneration in various congenital myopathies, such as Duchenne muscular dystrophy and congenital fiber-type disproportion	Cell therapy, exon skipping therapy, injection of MSCs directly into affected muscles, or systemic delivery of MSCs	[55, 56]
Regenerative medicine	MSCs are used as a potential therapy for muscle regeneration in various conditions	MSC-based tissue engineering, transplantation of exogenous MSCs	[57, 58]

SM regeneration and development is highly conserved in many species, including humans and model animals. Nonetheless, there are significant differences between the species. MSCs of both mice and humans can self-renew, differentiate into myoblasts, and contribute significantly to SM growth and regeneration. MSCs from both species can participate in in vivo muscle regeneration due to their stem cell characteristics; however, there are significant differences between the two. Although PAX7 is the standard marker for MSCs in both species, mouse MSCs also contain CD34, c-Met, integrin α 7, m-cadherin, and nestin, whereas human MSCs do not [59]. Different markers are also used to characterize quiescent, activated, proliferating, and differentiating MSCs in mice and humans. Furthermore, in vitro studies have shown that both mouse and human MSCs can differentiate into osteogenic and adipogenic pathways [60]. SM regeneration mechanisms differ substantially between species. Different species use different mechanisms for muscle regeneration. While some species rely on myofiber dedifferentiation, others use satellite-like cells (SLCs), which are similar to MSCs found in vertebrates. Xenopus and other amphibians repair muscle by increasing the number of Pax7 + myogenic cells, whereas salamanders use dedifferentiated myofibers or SLCs, depending on the species [61].

### Stem cells for muscle regeneration

Muscle regeneration involves various cell types. Myogenic cells, MSCs, and pluripotent stem cells differentiate into muscle cells and promote muscle repair. On the other hand, non-myogenic cells, such as mesenchymal stromal cells, adipose-derived stem cells, bone marrow-derived stem cells, and pericytes, reduce inflammation, and support muscle repair by secreting growth factors (GFs) and cytokines [43, 62]. Fibroblasts and endothelial cells also have potential muscle regenerative properties [63], and it now appears that combining multiple cell types with different regenerative properties offers a promising means of treating muscle-related disorders, including muscular dystrophy and myopathies.

#### Muscle satellite cells (MSCs)

MSCs, also known as adult SM stem cells, reside beneath the basal lamina near muscle fibers [60]. SM has a remarkable potential for regeneration. Even after several rounds of damage, the MSC pool remains constant, which demonstrates that the MSC population is self-renewing [64]. MSCs are stimulated upon receiving a signal of injury, leading to the secretion of a range of pro-inflammatory cytokines, namely interleukin-6 (IL-6) and interferon-gamma (IFN-γ). These cytokines play a crucial role in activating, proliferating, and facilitating the migration of MSCs [65]. Tumor necrosis factor (TNF-α) exerts distinct regulatory effects on MSCs based on their varying levels of expression. Specifically, when TNF-α is present at high levels, it facilitates the processes of proliferation and migration, while concurrently impeding the process of differentiation. The levels of TNF-α exhibit a decline as the inflammatory response subsides, while MSCs initiate the process of differentiation and subsequently generate new muscle fibers [66, 67]. The initiation of the inflammatory response in damaged muscle is triggered by the secretion of chemotactic factors, such as monocyte chemotactic protein 1, macrophage-derived chemokine, fractalkine, urokinase-type plasminogen activator/urokinase-type plasminogen activator receptor (uPA/uPAR), and vascular endothelial growth factor (VEGF), by MSCs [68, 69].

Furthermore, activated MSCs multiply and move to sites of injury to produce myoblasts [70] (Fig. 2), which then fuse to injured myofibers or combine to produce myotubes that mature to form new muscle fibers [71]. The PAX genes and MRFs, such as MyoD, Myf5, MyoG, and Myf6, primarily regulate MSC proliferation and differentiation during myogenesis, and sequential activations and repressions of Pax3/7 and MRFs are required for myoblast progression through myogenesis. All MSCs express Pax7, which is vital for the self-renewal and postnatal maintenance of these cells [72].

MSCs are essential for the natural regeneration of muscles after damage caused by exercise, injury, or disease. Stem cell therapy utilizing these cells is a promising treatment for muscle-related disorders, such as muscular dystrophies. Table 1 presents a comprehensive summary of the regenerative function of MSCs in different conditions and diseases, outlining their therapeutic potential for muscle regeneration.

#### Mesenchymal stem cells (MeSCs)

MeSCs are multipotent stem cells that generate mesodermal cells like osteoblasts, adipocytes, and chondrocytes [73], and several investigations have shown that MeSCs are involved in SM regeneration [74, 75]. However, the efficacies claimed for the myogenic differentiation of human MeSCs are debatable. Bone marrow-derived MeSCs are a type of multipotent, nonhematopoietic adult stem cell that can also be used for SM regeneration due to their myogenic potential [76]. For example, they are capable of differentiating into myoblasts in myogenic medium, or when co-cultured with myoblasts [71, 77], and after local (intramuscular injection) or systemic delivery (intravenous/intra-arterial injection), bone marrow-derived MeSCs have been reported to contribute to myogenesis [78–80]. Based on these findings, MeSCs have attracted interest in the regenerative medicine field over the last decade, particularly for SM regeneration, due to their advantageous properties, ready availability, multipotency, and active paracrine activity.

#### Adipose-derived stem cells (ADSCs)

ADSCs can differentiate into osteocytes, adipocytes, neuronal cells, vascular endothelial cells, myocytes, pancreatic cells, or hepatocytes and have morphological and immunophenotypic properties comparable to MSCs [81]. In myogenic media, ADSCs differentiated into myoblasts, as evidenced by the expressions of MyoD and myosin-heavy chains. Furthermore, pretreatment of ADSCs with IL-4 and stromal cell-derived factor-1 improved their myogenic ability [82]. ADSCs have shown remarkable promise for SM regeneration, and clinical trials have demonstrated their efficacy in enhancing muscle regeneration and function [83]. In addition, studies on mouse models of muscular dystrophy have shown that ADSCs can differentiate into SM cells and effectively treat COL VI deficiency [84].

#### Pericytes

Pericytes are perivascular stem cells found in the walls of capillaries and microvessels. These multipotent cells can differentiate into various cell types, including adipogenic, chondrogenic, and myogenic cells. Pericytes have been isolated from adipose tissue, pancreas, and SM [42, 73, 85] and, like MSCs, exhibit high myogenic ability in vitro and in vivo after muscle injury or in the presence of muscular dystrophy [42, 85, 86]. Pericytes in SM are multipotent and can differentiate into either the myogenic or adipogenic lineage [85]. Birbrair et al. found that type-1 pericytes contribute to fat infiltration in SM during muscle degeneration/regeneration, whereas type-2 pericytes form muscle and not fat after injury [85]. Muscle pericytes play crucial roles in the maintenance of myofiber size and stem cell quiescence, and when intra-arterially injected into dystrophin-null mdx mice colonized host SM and produced dystrophin-positive muscle fibers [42].

#### Induced pluripotent stem cells (iPSCs)

iPSCs can be produced in vitro by adding reprogramming elements, referred to as Yamanaka factors, to somatic cells. iPSCs can develop into practically any type of cell and have an unlimited potential for self-renewal in culture [87, 88]. Therefore, the ability of these cells to develop into myogenic cells makes them a desirable choice for myogenic regeneration [73, 89, 90]. Pax7 or MyoD overexpression can induce the differentiation of iPSCs into myogenic cells [91], and various studies have investigated the production of functioning SM in vitro by promoting the myogenic differentiation of iPSCs and fusing these cells with existing myofibers after in vivo transplantation [92–95]. In another study, human fibroblasts were used to produce iPSCs, and then Pax7 was activated to cause iPSC differentiation into myogenic progenitors with the ability to grow in vitro [96]. Table 2 summarizes the functions of SM regeneration stem cells.

**Table 2:** List of SM regeneration stem cells with detailed descriptions of their functions

Stem cells used for SM regeneration	Source	Specific role in SM regeneration	Advantages	Limitations	Reference
MSCs	SM tissue	SM-specific stem cells differentiate into myoblasts and fuse with prevailing muscle fibers to repair and regenerate SM tissues	High potential for muscle differentiation and self-renewal	Limited availability, potential for aging-related function decline	[97, 98]
Mesenchymal stromal cells	Bone marrow, adipose tissue, SM tissue	Promote angiogenesis, regulate inflammation, and promote cell differentiation into different cell types, such as myoblasts and myotubes	Low immunogenicity, can differentiate into multiple cell types including SM cells	Limited proliferation potential, the potential for heterogeneity	[68, 99]
Embryonic stem cells	Embryos	Pluripotent cells that can differentiate into all cell types, including muscle cells	High potential for differentiation and proliferation, can generate all cell types	Ethical concerns, the potential for teratoma formation, immune rejection	[100]
Induced pluripotent stem cells	Adult cells (e.g. skin cells) reprogrammed to pluripotency	Capable of differentiating into a wide range of cell types, including muscle cells, and can be derived from a patient's cells for personalized regenerative therapy	Possibility of personalized medicine, no ethical issues	Low efficiency of reprogramming, potential for genetic abnormalities, immune rejection	[90, 101]
Adipose-derived stem cells	Adipose tissue	Can differentiate into myogenic cells and aid in SM regeneration, as well as secrete trophic factors that promote muscle growth	Can differentiate into different types of cells, such as SM cells	Limited availability, the potential for heterogeneity	[84]
Pericytes	Blood vessels, SM tissue	Possess the capacity to differentiate into myogenic cells, helping to regenerate SM			[85]
Fibro-adipogenic progenitors (FAPs)	SM tissue	FAPs are activated during the regeneration process and migrate to the site of injury, where they differentiate into myofibroblasts and adipocytes	It can differentiate into muscle cells and regulate SM regeneration		[102, 103]

### Volumetric muscle loss

VML is a complex and heterogeneous type of SM injury caused by the surgical or traumatic excision of SM, as is commonly observed after chronic trauma [104]. Reduced muscle volume leads to the loss of contractile myofibers and depletion of the MSC reservoir, and this decline in MSCs at injury sites is accompanied by worsening muscle fibrosis, which reduces the ability of muscle to repair and regain contractile function [105, 106]. Currently, no standard treatment is available for completely replacing trauma-related VML. However, MSCs proximate to muscle fibers aid long-term SM maintenance and are activated in response to stimuli such as physical damage or growth signals. Following activation, MSCs divide symmetrically to increase their numbers or asymmetrically to create progenitor cells. Furthermore, myogenic progenitors undergo proliferation and subsequent differentiation through fusion under certain conditions to repair damaged fiber integrity and function. However, this ability is limited to a certain extent [97].

Several other therapies, such as tissue engineering [107], biological scaffolds composed of naturally occurring ECM [107, 108], hydrogels [109], immune response activation [110], cell transplantation [111], autologous grafting [112], scar tissue debridement, and minced skeletal tissue transfer [113], have been reported to repair VML. Natural polymers such as alginate, collagen (COL), and fibrin have been widely used in SM engineering [114, 115], and ECM molecules, especially COL, which acts as a reservoir for GFs, may also provide GFs to injury sites and increase muscle cell migration [116]. When incorporated into tissues, fibrin gels can increase myoblast survival and differentiation into myofibers [117]. In mouse models, fibrin scaffolds with a micro-thread architecture were also found to repair VMLs [118]. Furthermore, some medications like formoterol improved the strength and metabolism of VML-injured muscle [119], and in another study, treatment of VML with a fibrin hydrogel containing 450 g/mL of laminin-111 (FBN450) promoted muscle regeneration [120].

Regenerating SM to completely repair VML is a difficult and complex procedure that requires several stages. Many researchers have used an in vitro tissue culture phase to increase myoblast proliferation and achieve the functional maturity of SM constructs. Several approaches have been used to promote SM regeneration, including GFs, co-culture with supportive cell types, mechanical stretching, and electrical stimulation [121]. Numerous GFs are involved in muscle reconstruction and help to develop functional scaffolds by increasing myocyte contractility [45, 71, 122], fibroblast growth factor [123], prostaglandin E2 [124], hepatocyte growth factor [118], insulin-like growth factor [125, 126], platelet-derived growth factor, and Notch signaling are all important for MSC proliferation. Moreover, pro-angiogenic agents, including vascular endothelial growth factor, have been used to promote the vascularization of SM constructs [127]. Researchers appear to be focusing on the use of new biomaterials and tailored ECM scaffolds to enhance muscle regeneration after VML.

### Skeletal muscle ECM

The ECM is a network of structural molecules that facilitates biochemical signaling and provides mechanical support for cells, and thus, plays an essential role in regulating cellular proliferation, migration, and differentiation by interacting with cell surface receptors [10]. SM ECM is characteristically composed of COLs (predominantly COL IV and VI), fibronectin (FN), laminins, and others [128]. These ECM components and their specific receptors, such as integrin α7β1 and dystroglycan, play crucial roles in the development and maintenance of SM homeostasis [11]. A variety of muscle-related disorders, such as muscle dystrophy, are caused by deformities or mutations in ECM proteins, which can interact with MSCs and influence MSC activation, self-renewal, proliferation, and differentiation [129]. The proper functioning of MSCs is dependent on the regulation of the SM ECM, and therefore, any changes in ECM makeup can substantially alter the behavior of MSCs, which demonstrates that SM ECM and its contents are essential for the proper regulation of MSCs [130].

### ECM in muscle pathophysiology

ECM acts as a scaffold and helps organize muscle fibers into distinctive parallel arrangements that confer muscle strength and contractile ability. In addition, ECM contains molecules that support blood vessel formation, immune cell recruitment, and molecules that control muscle growth and repair [10, 131]. The ECM is composed of approximately 300 proteins that are collectively referred to as the core matrisome, which is composed of 43 COL subtypes, 36 proteoglycans, and nearly 200 complex glycoproteins [132]. This matrisome maintains ECM homeostasis, which is essential for individual cell function and cell-to-cell communication in a coordinated and systematic manner, and if this balance is disturbed, organ system functioning can be negatively impacted and the risk of severe diseases, including fibrotic diseases and cancer, increased [133]. Interestingly, it has been reported that certain genetic muscle-related diseases are primarily caused by mutations in ECM components and their receptors. In fact, over 150 ECM molecules are known to interact with the adhesion site of the integrin receptor [134, 135].

### Key constituents of SM ECM

SM ECM is a complex structure composed mainly of COLs, laminin, FN, and proteoglycans, which are crucial for the development, function, and physiology of SM [136, 137]. SM ECM is composed of three distinct layers epimysium, perimysium, and endomysium. The perimysium enfolds bundles of muscle fibers that originate in the epimysium, a dense connective tissue that surrounds entire muscles. Each muscle fiber is surrounded by a specialized membrane known as the endomysium, also referred to as basal lamina [138]. These three layers contain specific ECM molecules, viz. COL-1, undulin, tenascin, and FN in epimysium, COL-IV, laminin, FN, PGs, growth factor, and nidogen in endomysium, and COL-I, III, V, and VI in perimysium [136] (Fig. 2).

COLs are the most abundant type of SM ECM protein and account for 1 to 10% of SM dry weight and provide a structural framework for ECM. COL fibers are organized in a parallel manner that enhances SM strength and contractile ability [9, 136]. There are 28 forms of COL, and 11 of these have been identified in mature SM and are expressed during SM development [139]. COL VI regulates MSC self-renewal and SM regeneration and has been shown to regulate MSC activity by modulating muscle stiffness [140].

Laminins represent a glycoprotein family that plays a crucial role as fundamental constituents within the basement membranes [141]. Laminins are composed of a combination of five alpha (α) chains, three beta (β) chains, and three gamma (γ) chains, which collectively assemble into diverse heterotrimeric isoforms [141]. They provide mechanical support, enhance muscle cell-to-ECM adhesion, and can control muscle development and repair [136]. The typical expression of distinctive subtypes of laminins facilitates the process of regenerating damaged SM. Laminin-1 has the ability to maintain the connection between muscle fibers and the basal lamina, enhance muscle function in mdx mice, alleviate degeneration and inflammation in SM, expedite the process of muscle regeneration, and facilitate the proliferation and migration of myoblast cells [136, 142].

FN participates in ECM organization and the tethering of ECM to underlying muscle cells by integrin receptors [143]. Laura Lukjanenko et al. revealed that a decrease in FN levels in the SM milieu of elderly mice impairs muscle stem cell activation and maintenance via alterations in integrin-mediated signaling [144]. We recently identified FN-derived short peptides (FNIN 2 and FNIN 3) that promote cell adhesion, proliferation, the differentiation of primary and stem cells, and MSC viability in vitro [145].

Earlier, we investigated the roles of FMOD, DPT, and MGP in SM at various stages of development and during SM regeneration. FMOD plays a crucial role in the maintenance of ECM and facilitates muscle regeneration by increasing the recruitment of MSCs to sites of injury. FMOD is upregulated after muscle injury promotes new blood vessel formation and immune cell recruitment, and regulates ECM remodeling by regulating enzyme activity [16, 146]. DPT, another ECM protein, plays a role in the myogenic process by increasing cell adhesion and muscle differentiation while decreasing cell proliferation. DPT and FN inhibit each other in the myogenic setting, whereas DPT and FMOD positively regulate each other and promote muscle differentiation [20]. The observed decrease in myogenic marker and ECM gene expression in MGP knockdown cells suggests that MGP is involved in the regulation of myogenesis. Furthermore, the reduction in myostatin expression suggests that MGP may play a role in coordinating the control of myostatin expression [19].

### ECM in SM regeneration

It has been well established that ECM plays important roles in SM regeneration by serving as a scaffold for muscle cells to attach, proliferate, and transform in response to muscle injury or disease [135], and intact ECM can aid muscle fiber regeneration in damaged SM. Muscle fiber injury initially causes ECM hyperplasia, which increases SM tissue stiffness and acts to prevent further damage due to coordinated deadhesion and fibrosis. As MSCs differentiate, ECM is remodeled, and adhesion protein expressions are increased [147], whereas in cases of SM injury or myopathy, genes associated with ECM remodeling are upregulated, for example, metalloproteinases (MMP-2 and MMP-9), FN, Tenascin-C [136]. The coordinated expression of MMP-2 and MMP-9 is associated with distinct phases of the muscle degeneration and regeneration process. MMP-9 has been found to play a role in the activation of MSCs and the initiation of the inflammatory response, whereas MMP-2 activation has been associated with the regeneration of new myofibers [148, 149]. Expression of FN is transiently elevated during tissue remodeling and is predominant during embryonic development [150]. Following muscle injury, there is an increase in the expression of Tenascin-C in both injured and regenerating SM. This protein is believed to promote migration, inhibit premature fusion, and decrease cell adhesion [151].

Minor muscle injuries can be repaired by MSC-mediated regeneration, but extensive damage like VML and myopathies can lead to permanent reductions in muscle mass and function due to impaired regenerative capacity. Although cell-based therapies, such as autologous muscle transplantation and the injection of ex vivo cultured muscle cells, are promising treatment modalities for severe muscle injury and myopathy, their effectiveness is limited due to low transplanted cell survival rates [152]. Tissue engineering approaches to SM regeneration using rationally designed biomaterials have the potential to overcome the limits of conventional therapies [153]. Researchers have developed various biomaterials and scaffolds that mimic ECM and can be used to stimulate the proliferation and differentiation of stem cells into muscle cells to preserve SM regeneration. These biomaterials and scaffolds, which are primarily composed of ECM proteins such as COLs, laminins, and FN, can provide a supportive environment for stem cells to differentiate into muscle cells and facilitate their integration into host tissue [154–156].

### ECM scaffolds for SM repair

ECM scaffolds are three-dimensional biodegradable structures that mimic natural cellular environments and are frequently employed in tissue engineering and regenerative medicine to increase cell growth and proliferation and new tissue development [157]. ECM scaffolds have been used to promote SM repair due to their natural ability to facilitate cell infiltration and matrix remodeling, which are necessary for tissue regeneration [158]. Various ECM components, including as purified COLs, FN, fibrin, laminin, hyaluronic acid, and others, have been employed in the development of scaffolds. Scaffolds constructed from purified COL-1 are widely used as pure ECM components and have been approved by the FDA for several therapeutic applications [159].

#### Decellularized ECM Scaffolds

Decellularized extracellular matrix (dECM) scaffolds are biomaterials derived from human or animal tissues or organs after removing immunogenic cellular components using decellularization techniques and have emerged as a promising scaffold type in the SM tissue engineering field [160, 161]. dECM refers to the removal of cellular components from ECM to leave behind a water-insoluble matrix. dECM scaffolds closely resemble native tissue environments and promote tissue healing more than standard biomaterials, which makes them an appealing option for SM tissue engineering [162]. In surgical practice and regenerative medicine, the biocompatibility, biodegradability, and bioinductivity of dECM are critical considerations. Although dECM can be obtained from various organs and tissues, it is most typically derived from bone, breast, skin, or bladder. Furthermore, the utilization of whole organ decellularization to produce scaffolds for regenerating functional organs after cell loading has gained popularity recently [163–165].

The main components of dECM are COL, elastin, FN, laminin, and matricellular proteins. dECM scaffolds are classified into autogenous, allogeneic, and xenogeneic types based on their origins. Because tissue availability is limited and autogenous dECM scaffolds often present surgical challenges, allogeneic or xenogeneic donor tissues are commonly utilized to produce dECM scaffolds. On the other hand, the use of allogeneic or xenogeneic dECM scaffolds may result in donor site morbidity and differences in architecture and mass composition. Incomplete decellularization may also cause immunogenicity issues for dECM scaffolds [161, 166].

### Biomimetic scaffolds for SM regeneration

In the recent era of muscle regeneration, particularly for severe muscle loss or VML therapy, research has focused on the use of suitable biomaterials as templates to guide tissue reorganization and provide optimum micro-environments for cells [14]. Biomimetic scaffolds have the potential to increase SM regeneration by providing a framework for stem cell proliferation and differentiation, stimulating the development of new blood vessels, and integrating new and existing tissues [167]. This strategy has the potential to address the limits of the body's inherent ability to rebuild muscle tissues, and researchers are creating scaffold-based solutions to boost the body's ability to mend and regenerate injured SM tissues using the principles of biomimicry. The following are some of the most commonly used ECM scaffolds for muscle regeneration and tissue engineering.

#### Hydrogel-based scaffolds

Hydrogels are made up of 3D networks that absorb a lot of water without dissolving in an aqueous media. This fundamental property of hydrogels promotes scientific study interest in a dominant path in extending their potential in a variety of sectors [168]. Naturally, hydrogels are suitable candidates for in vivo applications because they have low inflammatory responses, are composed of structural components similar to those found in the body, and are effective at initiating SM regeneration [169, 170]. The materials explored for SM tissue engineering include COL, gelatin, fibrin, Matrigel, keratin, hyaluronic acid, silk, and alginate-based hydrogels [171, 172]. Synthetic hydrogels are less favored for SM tissue formation than natural polymer-based hydrogels. However, synthetic polymer-based hydrogels can be designed to enable the controlled release of GFs to stimulate muscle regeneration. The main limitations of these hydrogels are low cell adhesion (as compared with natural hydrogels) and the risk of foreign body reactions due to the polymer used or its degradation products [173, 174].

#### Nanofibers

Polymeric nanofibers are excellent vehicles for the release of bioactive chemicals and substances, such as GFs, medicines, herbal extracts, and gene sequences, due to their high surface-to-volume ratios [175]. Nanofibers are employed in various biomedical applications, including tissue engineering, medical implants, antimicrobial barriers, and wound dressings [176]. Cagla Eren Cimenci et al. reported that laminin-mimetic peptide nanofibers promoted the activation of MSCs and their myogenic differentiation in vivo and that therapy using these nanofibers increased SM regeneration by boosting satellite cell recruitment and muscle fiber expansion [177].

#### Electroconductive scaffolds

By providing physical and electrical support for the growth of new muscle tissue, electroconductive scaffolds have the potential to improve the outcomes of muscle regeneration therapies [178]. Electrical impulses are generated by motor neurons and cause voluntary muscle contractions. To support the growth of new muscle tissue, these impulses must be generated by applying an external source to electrically conductive scaffolds in vitro. Electrical stimulation of cells seeded on conductive scaffolds made of polyurethane-carbon nanotubes, for example, has been demonstrated to promote the adhesion and differentiation of C2C12 cells and stimulate the production of myotubes [179]. Selva Bilge et al. created aligned, 3D-printed, electrically active scaffolds using a carbonaceous material (CM) produced by the hydrothermal carbonization of an algae-based biomass. During in vitro culture, scaffolds were seeded with C2C12 mouse myoblasts, and electrical stimulation was applied. The authors found that electrical stimulation resulted in more myotube production and that hydrothermal carbonization accelerated myotube maturity [180]. Xiaoyan Tang et al. reported the fabrication of a novel stimulus-responsive conducting polymer scaffold that regulated muscle cell adhesion, proliferation, and differentiation [181]. These biomimetic platforms for SM regeneration hold much promise for the future.

#### 3D graphene scaffolds

Because of their unique interactions with proteins and molecules, graphene-based materials (GBMs) have been investigated for various biomedical applications. The surface area and chemistry of GBMs result in strong protein adsorptions that can mediate interactions between delivered medicines and bacteria or host cells [182, 183]. Scaffolds for SM regeneration must have specific properties that direct myocyte fusion with multinucleated myotubes and induce vascularization and innervation. Furthermore, when the regenerated tissue is established, materials must decay in a biocompatible manner [8].

### Limitations of ECM scaffolds for SM repair

Despite the advantages of ECM scaffolds, such as their ability to induce constructive remodeling of injured tissues, their efficacy in restoring SM structure and function following injury is limited by inherent limitations [159]. One of the main challenges associated with the utilization of different biological scaffolds, particularly those derived from dECM components, is the inadequate alignment of regenerating tissue with pre-existing healthy tissue. Although the use of dECM materials has demonstrated notable advancements in addressing VML deficiencies, achieving seamless integration between newly generated tissue and pre-existing healthy tissue continues to be an ongoing obstacle [14].

Natural scaffolds outperform typical synthetic materials in terms of promoting tissue regeneration, notwithstanding their intrinsic bioactivity. In contrast, the ability to achieve consistent cellular alignment is lacking when large-scale scaffolds are generated using uncontrolled protein polymerization methods. This disparity complicates achieving full SM recovery because it necessitates extensive regeneration at both the functional and structural levels [8]. To fully harness the therapeutic capabilities of ECM scaffolds, extensive research is required to devise novel scaffolding technologies that surpass current limitations and facilitate the advancement of more effective strategies for muscle repair and regeneration.

### Concluding remarks

Muscle regeneration and myogenesis are intricate processes that involve the coordinated collaboration of various stem cell populations and ECM components. By gaining a deeper understanding of this complexity, more precise interventions that aid and amplify the innate capacity of the body to regenerate injured SM tissue can be devised. To more fully comprehend the potential of stem cell transplantation for muscle regeneration, it is important to identify mechanisms responsible for better muscle repair and function and to understand the regulatory processes that govern the differentiation of diverse stem cell types, including satellite and non-satellite cells, during muscle regeneration.

Extensive muscle damage like VML and myopathies can lead to permanent reductions in muscle mass and function due to impaired regenerative capacity. Tissue engineering has advanced significantly over the last decade and is expected to provide therapies for SM regeneration. Biomaterials are required that address the current limitations of conventional therapies, and biomaterials and scaffolds typically composed of ECM proteins, such as COL, laminin, and FN, that mimic natural ECM, have been shown to support the growth and differentiation of stem cells into muscle cells. In addition, novel technologies are needed to boost biomaterial properties and improve stem cell-mediated and ECM-supported muscle regeneration therapies. This review offers an overview of the muscle regeneration process and highlights the importance of ECM in the search for innovative biomaterials for tissue engineering. In addition, we hope this review encourages the development of more effective muscle repair and regeneration strategies, advances the field of muscle regeneration, and improves the quality of life of those suffering from muscle injuries and disorders.

## Data Availability

Not applicable.
